# Impact of national influenza vaccination strategy in severe influenza outcomes among the high-risk Portuguese population

**DOI:** 10.1186/s12889-019-7958-8

**Published:** 2019-12-16

**Authors:** Ausenda Machado, Irina Kislaya, Amparo Larrauri, Carlos Matias Dias, Baltazar Nunes

**Affiliations:** 1 Departamento de Epidemiologia, Instituto Nacional de Saúde Doutor Ricardo Jorge, Av. Padre Cruz, 1649-016 Lisbon, Portugal; 2 NOVA National School of Public Health, Public Health Research Centre, Universidade NOVA de Lisboa, Lisbon, Portugal; 3 National Centre of Epidemiology, Institute of Health Carlos III CIBER de Epidemiología y Salud Pública (CIBERESP), Madrid, Spain

## Abstract

**Background:**

All aged individuals with a chronic condition and those with 65 and more years are at increased risk of severe influenza post-infection complications. There is limited research on cases averted by the yearly vaccination programs in high-risk individuals. The objective was to estimate the impact of trivalent seasonal influenza vaccination on averted hospitalizations and death among the high-risk population in Portugal.

**Methods:**

The impact of trivalent seasonal influenza vaccination was estimated using vaccine coverage, vaccine effectiveness and the number of influenza-related hospitalizations and deaths. The number of averted events (NAE), prevented fraction (PF) and number needed to vaccinate (NVN) were estimated for seasons 2014/15 to 2016/17.

**Results:**

The vaccination strategy averted on average approximately 1833 hospitalizations and 383 deaths per season. Highest NAE was observed in the ≥65 years population (85% of hospitalizations and 95% deaths) and in the 2016/17 season (1957 hospitalizations and 439 deaths). On average, seasonal vaccination prevented 21% of hospitalizations in the population aged 65 and more, and 18.5% in the population with chronic conditions. The vaccination also prevented 29% and 19.5% of deaths in each group of the high-risk population. It would be needed to vaccinate 3360 high-risk individuals, to prevent one hospitalization and 60,471 high-risk individuals to prevent one death.

**Conclusion:**

The yearly influenza vaccination campaigns had a sustained positive benefit for the high-risk population, reducing hospitalizations and deaths. These results can support public health plans toward increased vaccine coverage in high-risk groups.

## Background

The annual circulation of influenza virus causes epidemics that can lead to a considerable burden of hospitalization and death, especially for a sub-group of the population with a high risk of influenza complications. It has been estimated that annually influenza is responsible for an excess of respiratory deaths that ranged from 4.0 to 8.8 per 100,000 worldwide, and these figures increased with age, causing an excess of 2.9 to 44.0 per 100 000 individuals in the 65 to 74 age group [1]. In Portugal, influenza burden patterns are similar, and in the all-age population, influenza epidemics have been estimated to be associated with an average of 24.7 all-cause excess deaths per 100,000 [2] and 19.4 per 100,000 excess pneumonia and influenza hospitalizations [3].

Besides age, the presence of certain medical conditions, namely diabetes, obesity, immunodeficiency and chronic respiratory, cardiovascular, kidney and renal diseases, are well-established risk factors for severe influenza complications such as hospitalizations, intensive care and death [4].

In Portugal, an at-risk based vaccination program has been in place at least since 2001/2002. In accordance with the National Directorate for Health clinical guidelines [5], influenza vaccination is strongly recommended to those at higher risk for influenza complications (chronic and immunocompromised patients older than 6 months of age, pregnant women), as well as those aged 65 and over and to institutionalized to whom the vaccine is offered free of charge. Seasonal influenza vaccination is also recommended to health professionals and other caregivers.

Every year seasonal influenza vaccination campaigns with inactivated trivalent vaccine start in early October run throughout the fall and winter. Despite gradual increase in influenza vaccine uptake in the 65 years and older population [6], in Portugal, like in most other European Union (EU), vaccination coverage (VC) still has not reached the target of 75% set by the World Health Organization and the European Commission (EC) [7]. Individuals with chronic conditions have even lower seasonal influenza vaccine coverage’s, that ranged between 32.3% in 2015/16 and 41.0% in the 2017/18 season [8].

To increase the acceptability of the vaccine, it is important to quantify the benefits of annual influenza vaccination, namely by estimating its impact at the population level. Influenza vaccine effectiveness (IVE) studies are conducted in Europe every season and allow early and end of season estimates on the reduction of medically attended confirmed influenza infections, either at primary care or hospital level [9–11]. However, population data on influenza-associated outcomes prevented each season by influenza vaccination, in high-risk populations, is scarce. Moreover, measuring the impact of the influenza vaccination strategy every season is methodologically challenging, as influenza vaccination programmes are in place for several decades, which inhibit the before/after comparison of the introduction of this public health intervention. To overcome this, some authors use an ecologic approach that permits the estimation of influenza vaccination impact using data on vaccine coverage, vaccine effectiveness and the number of observed influenza events [12–16].

This study aimed to estimate, the influenza-related hospitalizations and intra-hospital deaths attributable to influenza averted by seasonal influenza vaccination strategy, during the influenza seasons 2014/15 to 2016/17, in individuals 65 years and older and those, at any age, with comorbidity that represents a high risk group for influenza complications.

## Methods

### Hospitalization data

We developed an observational retrospective ecologic study using the National Hospital Discharge Database.

This database covers all public hospitals in Portugal mainland and includes demographic data, diagnosis, procedures, length of stay and discharge outcomes for all episodes of hospital care. In the 2006–2016 period, this database included approximately 79% of all hospital admissions that occurred in Portugal [17]. Diagnoses and some procedures (laboratory results not included) are coded using the International Classification of Diseases (ICD) - 9th Revision Clinical Modification (ICD-9-CM) version [18] until 1st January 2016 and ICD-10-CM [19] onwards. Final validated databases are available for research after every 9 to 12 months.

### Study population

The target population of this study is high-risk individuals, namely, aged 65 and older or < 65 years with a chronic condition (diabetes, chronic respiratory, cardiovascular, kidney and renal diseases, obesity, immunodeficiency) for which the influenza vaccine is recommended [20–22]. An individual was considered as having a chronic condition if it was hospitalized and had a secondary diagnosis within a set of chronic conditions presented in Table 1.

**Table 1:** List of International Classification of Diseases (ICD) 9th and 10th version codes for chronic conditions

	ICD 9th version	ICD 10th version
Respiratory	011, 490–511, 512.8, 513–517, 518.3,	A15, J40–47, J60–94, J96, J99, J182,
	518.8, 519.9, 714.81	M34.81, M05.10
Cardiovascular	746.9	Q24.9
	402.0–402.91	I11.0-I11.9
	428.42, 428.32, 482.22	I50.22, I50.32, I50.42
	412,0-412,9, 413.0–413.9, 414.0–414.9	I25.2, I20.8, I20.1, I20.9, I25.0-I25.9
Renal	581.0–581-9, 585.0–585.9	N18, N04
Kidney	571.0–571.9	K70, K74, K72.1
	576.2	
Hematologic	282.4, 282.5, 282.6	D56, D57
Imunocompromised	042, 279, V08, V42	B20, D80–84, D89.8–9, Z21, Z94
Diabetes mellitus	250	E10–11; Z94.0-Z94.4, Z94.6-Z94.9
Genetic conditions	273.4	E88.01
Obesity	278.00, 278.01, 278.03	E66.01, E66.2, E66.9

*ICD* International Classification of Diseases (ICD)

To estimate the number of high-risk individuals in Portugal, we used the population estimates from the national statistical office (Statistics Portugal) [23] and the proportion of self-reported chronic conditions in the 0–4 and 15–64 age groups from two health surveys representative at National and regional level [24, 25].

### Severe influenza-related events

We considered two severe influenza-related outcomes: hospitalizations (admissions for > 24 h) and intra-hospital deaths attributable to influenza.

#### Hospitalizations

Hospital admissions due to influenza were obtained by multiplying the weekly number of severe acute influenza respiratory illness (SARI) hospitalizations by the weekly proportion of SARI influenza positivity. The proportion of influenza-positive was obtained from the hospital-based Portuguese laboratory network for the diagnosis of influenza [26].

A SARI hospitalization was defined as an episode with hospital stay length higher than 24 h with a primary diagnosis coded as any of SARI codes defined in Integrated Monitoring of Vaccines Effects in Europe (I-MOVE+) protocol [27] (Table 2).

**Table 2:** List of diagnosis codes for which patients could be screened for onset of SARI symptom, IMOVE+ hospital based IVE studies

Category	Morbidity	ICD-9	ICD-10
Influenza like illness	Cough	786.2	R05
	Difficulty breathing	786.05	R06
	Sore throat	784.1	R07.0
	Dysphagia	787.20	R13
	Fever	780.6	R50.9
	Headache	784.0	R51
	Myalgia	729.1	M79.1
	Fatigue/malaise	780.79	R53.1, R53.81, R53.83
Cardiovascular diagnosis	Acute myocardial infarction or acute coronary syndrome	410–411, 413–414	I20–23, I24–25
	Heart failure	428 to 429.0	I50, I51
Respiratory diagnosis	Emphysema	492	J43.9
	Chronic obstructive pulmonary disease	496	J44.9
	Asthma	493	J45
	Myalgia	729.1	M79.1
	Dyspnoea/respiratory abnormality	786.0	R06.0
	Respiratory abnormality	786.00	R06.9
	Shortness of breath	786.05	R06.02
	Other respiratory abnormalities	786.09	R06.00, R06.09, R06.3, R06.89
Infections	Pneumonia and influenza	480–488.1	J09-J18
	Other acute lower respiratory infections	466, 519.8	J20-J22
	Viral infection, unspecified	790.8	B34.9
	Bacterial infection, unspecified	041.9	A49.9
	Bronchitis	490, 491	J40, 41
Inflammation	SIRS non infectious without acute organ dysfunction	995.93	R65.10
	SIRS non infectious with acute organ dysfunction	995.94	R65.11
Diagnoses related to deterioration of general condition or functional status	General physical deterioration, lethargy, tiredness	780.79	R53.1, R53.81, R53.83
	Anorexia	783.0	R63.0
	Feeding difficulties	783.3	R63.3
	Abnormal weight loss	783.21	R63.4
	Other symptoms and signs concerning food and fluid intake	783.9	R63.8
	Desorientation/Altered mental status	780.97	R41.0
	Dizziness and giddiness	780.4	R42
	Infective delirium	293.0, 293.1	F05
	Coma	780.01	R40.2
	Transient alteration of awareness	780.02	R40.4
	Other alteration of consciousness (Somnolence, stupor)	780.09	R40.0, R40.1
	Febrile convulsions (simple), unspecified	780.31	R56.00
	Complex febrile convulsions	780.32	R56.01

^*a*^*SIRS* Systemic inflammatory response syndrome

#### Deaths

Intra-hospital deaths attributable to influenza (from this point forward designated as deaths) were estimated using the hospital discharge outcome information. The number of deaths that occurred in patients hospitalized with SARI diagnosis during the study period was multiplied by the proportion of influenza-positive, obtained from the Portuguese laboratory network for the diagnosis of influenza [26].

### Study period

The impact o**f** the influenza vaccine national program was estimated for three seasons, 2014/15, 2015/2016 and 2016/17. For each season the analysis was restricted to the epidemic periods. Epidemic periods were established by the primary care-based Portuguese sentinel influenza surveillance system (Table 3). For each season, the study period comprises the epidemic periods, plus 3 weeks lag as more severe outcomes are expected to occur with delay.

**Table 3:** Distribution per season of the number of severe acute respiratory infections (SARI) hospitalizations and deaths, SARI Influenza-related hospitalizations and deaths, influenza vaccine effectiveness and vaccine coverage estimates for ≥65 years and < 65 years with a chronic condition

Season features	2014/15	2015/16	2016/17
Epidemic period	w1 to w7/2015	w53/2015 to w7/2016	w48/2016 to w1/2017
Dominant type/subtype	B/Yam	AH1N1pdm09	AH3
SARI Hospitalizations			
≥ 65 years (n)	24,720	21,913	18,724
Chronic condition (n)	4379	4595	3231
SARI deaths			
≥ 65 years (n)	3824	3044	2942
< 65 years with a chronic condition (n)	198	196	137
Influenza hospitalizations			
≥ 65 years	6742	5107	6003
< 65 years with a chronic condition	1204	1069	1031
Influenza deaths			
≥ 65 years	1078	731	909
< 65 years with a chronic condition	56	47	42
Influenza vaccine effectiveness (IVE %)			
≥ 65 years[95%CI]	29.0 [14.7 to 43.4]	49.1 [26.4 to 71.7]	42.9 [33.0 to 52.8]
< 65 years [95%CI]	46.1 [20.9 to 71.3]	53.0 [34.9 to 71.1]	58.9 [38.1 to 79.6]
Vaccine coverage (VC %)			
≥ 65 years[95%CI]	49.8 [41.3 to 58.4]	50.1 [42.1 to 58.1]	57.5 [50.8 to 64.1)
Chronic condition [95%CI]	34.2 [28.1 to 40.9]	32.3 [26.8 to 38.4]	39.7 [33.7 to 45.9]

*SARI* severe acute respiratory infections*, w* week*, IVE* influenza vaccine effectiveness*, CI* confidence intervals*, VC* vaccine coverage

### Number of averted severe influenza-related events prevented fraction

To assess the annual impact of the influenza vaccination programmes we estimated the number of severe influenza-related events (IRE) among the high-risk population averted by vaccination (NAE), the respective disease prevented fraction (PF) and the number of high-risk individuals in the population needed to be vaccinated to avoid one IRE (NNV).

NAE measures the impact of the vaccination program in absolute terms and represents the difference between observed IRE (n) and IRE expected in the absence of the vaccination program (N) and was estimated as:

$$ NAE=\mathrm{N}-\mathrm{n}=\mathrm{n}\bullet \frac{IVE\bullet VC}{1-\left( VC\bullet IVE\right)} $$

where n – observed IRE, IVE – Influenza vaccine effectiveness, VC – vaccine coverage (details on the formula available at Additional file 1).

The PF, estimated as PF = NAE/(n + NAE), measures the impact of vaccination in relative terms and represents the proportion of averted IRE out of the number of IRE in the population without influenza vaccination program [12–14, 16, 28].

The number needed to vaccinate was also computed as:

NNV = 1/(IVE·N/Population).

### Vaccine coverage (VC)

The influenza vaccination coverage was estimated using the influenza vaccine coverage monitoring system [6, 29], a population-based survey of a sample of approximately 1000 households from Portugal mainland, selected using random digit dialling of mobile and landline phones. In each household, one individual aged 18 or more is interviewed providing information on his/her vaccination status and the vaccination status of the other household elements. The questionnaire also includes information on chronic conditions. Vaccine coverage was estimated for the population with conditions for which the vaccine is recommended and for the population aged 65 and more years.

### Influenza vaccine effectiveness (IVE)

We used hospital-based meta-analysis type/subtype IVE estimates. IVE among those aged 65 and older and with less than 65 years [30] were weighted by the distribution of circulating influenza type/subtypes virus in each season in Portugal. Reported match/unmatched vaccine information [30] was considered. Data on circulating influenza type/subtypes detected in the hospital settings were obtained from the Portuguese Hospital laboratory network for the influenza diagnosis [26, 31].

Vaccine effectiveness against intra-hospital deaths of 56% [14 to 77%] reported by Casado et al. [32] for the Spanish population was used for all high-risk groups.

### Uncertainty

To estimate 95% confidence intervals for NAE, NNV, and PF we used Monte Carlo simulations (see more detail in Additional file2). We assumed a Poisson distribution for the number of influenza-related events and Normal distributions for log (1-IVE) and log (VC/(1-VC)) [13]. The distributions parameters were derived from respective point estimates and 95% confidence intervals. We drew 10,000 simulations samples of influenza-related events, IVE and VC to obtain empirical distributions of NAE, NNV, and PF in each season and average across three seasons. We used the 2.5 and 97.5 percentiles of these empirical distributions as lower and upper limits of the 95% confidence intervals.

## Results

We estimate that during the study period there were about 3.82 million high-risk individuals in Portugal targeted by the National vaccination program: 2.07million were aged 65 and more and about 1.75 million aged less than 65 years and had a chronic condition for which the influenza vaccine is recommended.

During the study period, the estimated number of SARI hospitalizations among high-risk individuals ranged from 21,955 (season 2016/17) to 29,099 (season 2014/15) (Table 3). Influenza deaths varied between 778 in season 2015/16 and 1134 in season 2014/15.

The most frequent comorbidities of hospitalized SARI patients were chronic respiratory diseases (46%), followed by diabetes (32%) and cardiovascular diseases (24%). The highest number of influenza-related hospitalizations and deaths was estimated in season 2014/15, a B/Yamagata dominant season but with A(H3N2) circulation (7946 hospitalizations and 1134 deaths). Influenza vaccine effectiveness was lower in the 2014/15 season than in the other seasons. Overall, point estimate IVE was lower in the individuals aged 65 and more years than in younger individuals with chronic conditions. In contrast, vaccine coverage was higher in the older population, increasing from 49.8% in the 2014/15 season to 57.5% in the 2016/17 season.

On average, per season, the influenza vaccination campaign averted approximately 1833 hospitalizations and 383 deaths in the Portuguese high-risk population (Table 4). The highest number of averted events occurred in the population aged 65 and more years and the 2016/17 season, a season with a predominance of A(H3N2) virus.

**Table 4:** Estimates of the annual average and seasonal prevented fraction and number of averted influenza hospitalizations and deaths attributed to the vaccine program, for seasons 2014/15 to 2016/17

IRE	Season			Average
	2014/15	2015/16	2016/17	
Hospitalization				
≥ 65 years				
NAE (n) [95%CI]	1124 [443–1863]	1644 [460–2799]	1957 [1326–2657]	1584 [1058–2097]
PF (%)[95%CI]	14.3 [6.2–21.6]	24.3 [8.2–35.4]	24.6 [18.1–30.7]	21.0 [15.1–26.1]
NNV (n)[95%CI]	890 [565–2233]	621 [384–2050]	616 [473–882]	741 [553–1426]
< 65 years with chronic condition				
NAE (n) [95%CI]	222 [42–383]	218 [106–326]	311 [135–473]	249 [158–337]
PF (%)[95%CI]	15.6 [3.3–24.1]	16.9 [9.1–23.4]	23.2 [11.6–31.5]	18.5 [12.5–23.4]
NNV (n) [95%CI]	2657 [1628–10,924]	2564 [1857–5172]	2200 [1540–4957]	2619 [1921–6015]
Deaths				
≥ 65 years				
NAE (n) [95%CI]	409 [81-721]	281 [52-489]	427 [95-743]	371 [198-541]
PF (%) [95%CI]	27.7 [7.0-40.1]	27.5 [6.7-40.1]	31.9 [9.4-45.0]	29.0 [18.0-37.4]
NNV (n) [95%CI]	2416 [1459-10,267]	3613 [2166-15,078]	2796 [1633-10,422]	3241 [1845-10,141]
< 65 years with chronic condition				
NAE (n) [95%CI]	13 [3–22]	10 [2–49]	12 [3–20]	12 [6–16]
PF (%)[95%CI]	18.9 [4.8–27.9]	17.9 [4.3–26.1]	22.0 [6.5–31.7]	19.5 [11.8–25.4]
NNV (n)[95%CI]	45,211 [29,413–176,520]	54,149 [35,348–205,119]	58,216 [37,146–206,237]	57,230 [37,610–176,457]

*IRE* influenza related event, *NAE* number of averted events, *CI* confidence interval, *PF* prevented fraction, *NNV* number needed to vaccinate

Overall, the vaccination strategy prevented on average, 21 and 18.5% of influenza hospitalizations in those 65 years and older and under 65 years with chronic conditions, respectively, and 19.5% and 37.4% of deaths in the < 65 years and in the older adult population, respectively. To prevent one influenza-related hospitalization or death, we would need to vaccinate on average 3360 and 60471 high-risk individuals, respectively.

## Discussion

We estimated that during the influenza seasons 2014/15, 2015/16 and 2016/17, the trivalent inactivated seasonal influenza vaccination strategy averted each season, on average, more than 1830 hospitalizations and 380 deaths in the high-risk population in Portugal.

Overall, the impact of the program was higher in the population aged 65 and more years, a sub-group with higher VC but systematically lower IVE, in which it accounted for 86 and 95% of the total number of averted hospitalizations and deaths. These results reflect not only the higher risk of complication of that population but also the potential gain that could be achieved by increasing the vaccine coverage. In seasons with lower influenza vaccine, performance high vaccine coverage could balance and allow reasonable prevented fraction. Also, it demonstrate that, even with limited effectiveness, the influenza vaccination program prevented a considerable number of hospitalizations and deaths associated with influenza in the 65 years and older population.

The results also show that, even in seasons with a mismatch, between circulating virus and vaccine composition, like the 2014/15 season, vaccinating this high-risk group of individuals averted 1346 hospitalizations and 264 deaths. This included averting 5% premature deaths of individuals aged < 65 years. The highest number of averted events occurred in the A(H3N2) dominant 2016/17 season, where approximately 2268 hospitalizations and 442 deaths were prevented. These results are important given that in seasons with A(H3N2) predominant circulation i) a considerable burden of influenza is observed in the target vaccination subgroup and ii) vaccine effectiveness tends to be low against this virus sub-type [30, 33]. Besides, in seasons with A(H3N2) predominance, the influenza vaccination impact is limited in preventing primary care consultations by influenza [34] and thus this result reinforces the main objective of the influenza vaccination strategy as the reduction of severe complications by influenza.

The estimated prevented fractions indicate that on average, per season, the influenza vaccination strategy prevented 21% of the hospitalization in the population aged 65 and more and 18.5% in the < 65 years with a chronic condition. Season specific estimates in 2015/16 season (PF = 24.3%) was comparable to estimates obtained in a study for the USA population for the same season (22.5, 95% CI: 13.5–31.4) [35] and were also comparable to estimates reported for the USA population in other seasons with predominant circulation of A(H1)pdm09 virus [36]. However, for the 2014/15 and 2016/17 season, our estimates were higher than the reported for the USA. For instance, it was double than the 7% estimates published for the 2014/15 season [15, 37] and 11.5% estimates for the 2016/17 season [38]. Considering that the PF mainly depends on the VC and IVE estimates [39] and that VC was comparable between countries and was stable along the period in study, the main contributor to the observed differences was most probably IVE. In our study, we used type/subtype specific meta-analysis estimates and weighed to account for the virus in circulation. In the 2014/15 season, although the mismatched A(H3N2) virus circulated in Portugal, the predominant virus was type B virus [40]. As such, season-specific IVE for 2014/15 season was estimated to be 29% in the more than 65 years population and 46% in the ones with chronic conditions, thus justifying the increased estimated PF. In the 2016/17 season, the predominant A(H3N2) in Portugal matched the vaccine strain and this study final IVE estimates for the population aged 65 and more were considerably higher than the 17% published in Europe [41] and 20% in the USA [38].

Concerning the most severe outcome, we estimate that 19.5% or 29% of hospitalized influenza deaths were prevented by the vaccination strategy. The comparison with other studies is limited as most studies focus on older adults and all-cause mortality [16] or cause-specific mortality outcomes [13, 15]. Nevertheless, and though using a conservative estimate of 56% reduction of influenza-related intra-hospital deaths, we estimate that the vaccination strategy would prevent premature deaths in < 65 years population with chronic conditions.

To prevent one hospitalization or death we would need to vaccinate additionally 3982 individuals aged 65 and more years and 59,849 individuals with chronic conditions. These results indicate that there is a potential increased benefit if the 75% VC target would be achieved.

The results presented should be interpreted in light of the study’s limitations. The ecologic nature of the study and the use of several data sources limits the study external validity.

In relation to the study design, the main objective of the study is to measure the impact of the influenza vaccination programme and this is methodologically challenging. For some infectious diseases, as pneumococcal pneumonia, the impact of vaccination in the older adult Portuguese population was measured by comparing hospitalizations rate before and after the introduction of the pneumococcal conjugated vaccines [42]. However, for influenza vaccination, with a vaccination programme in place for long time, such approach is not feasible. The method that we used to estimate the influenza vaccination strategy impact has been used by several countries [12–16, 28] and consists in evaluating how the vaccination programme works at population level, using as reference an hypothetical totally susceptible population that has never been exposed to the intervention.

The methods measure only the direct effect of vaccination in the population and thus represents a more conservative estimate of the impact as does not account for indirect effects. Also, there are some limitations to the data. First, hospitalization and deaths were retrieved from a hospital discharge database collected for administrative and hospital financing purposes, which covers approximately 79% of the national hospitalizations [17]. Another limitation of this database to estimate the impact in each season is the time to have available data. The hospitals are in the process of changing from ICD9 to ICD10 and this has delayed obtaining the final database jeopardizing the use of annual estimation of vaccination strategy impact to prepare the next season. Finally, this database includes diagnoses and some procedures codes, however, laboratory results are not systematically done nor available which prohibit the use of influenza laboratory diagnoses directly from it. This limitation had as consequence the need of using an external database (from the hospital-based Portuguese laboratory network for the diagnosis of influenza) to obtain an estimate of the influenza hospitalized cases.

In our study, the VC was obtained from self-reported data and restricted to non-institutionalized Portuguese mainland residents. Given that VC is higher in the institutionalized population, our VC results may be underestimated and thus underestimation of impact results. Finally, IVE against mortality may not represent the Portuguese population, since we used VE derived from a study in a population from Spain that could have a different distribution of chronic conditions and access to health care. Also, the same estimate was used for all seasons and sub-group of population and IVE is expected to vary between seasons and in younger individuals.

The study has also considerable strengths. First, to our knowledge, this is the first attempt to measure the vaccination impact in a population < 65 years with chronic conditions. Considering that this subgroup is targeted by influenza vaccination programs and is an important fraction of the population (26.2% of population aged 15–64 years) our results demonstrate important benefits by the seasonal vaccination in this sub-group. In a country with low seasonal adherence to influenza vaccine uptake in the group of the population with a chronic condition for which seasonal influenza vaccination is recommended [43], this information could be important to increase vaccine coverage in this target group. Second, we used an alternative method to estimate influenza severe burden in Portugal to better fit the impact study. Given the ecologic nature of the adopted approach, it was important to have specific outcomes and also that had correspondence to the outcome of IVE estimates.

Previous research in Portugal provided estimates of influenza excess associated hospitalizations [3] and all-cause deaths [2] that were based on time series approaches. Although these methods are more comprehensive approaches and often used to measure influenza burden [44], in our case, it was important to use specific influenza-related outcomes for impact estimation. We used hospitalized SARI and intra-hospital deaths, restricted to epidemic periods, to improve specificity. Also, it was important to have an outcome that was highly correlated with IVE estimates. Finally, to increase external validity we used i) a national discharge database, ii) an influenza laboratory diagnoses database that collects influenza positivity results from hospitals distributed at national level; iii) influenza vaccine effectiveness from meta-analysis and iv) vaccine coverage estimates from a population-based survey. Moreover, to reduce potential heterogeneity related to different codification procedures, the study was restricted to only 3 seasons, but that with different pattern of the influenza virus circulation. All these using registry/ monitoring data easily accessible that can be replicable each season. Following the example of the USA [45], these results, along with the burden and effectiveness results can be reported, so to better communicate the influenza vaccination benefits.

## Conclusion

The influenza vaccination strategy in place in Portugal for the 65 and more years individuals and individuals with chronic conditions, prevented on average 1833 hospitalizations and 383 deaths per season. The applied method identified and quantified the overall benefits of the influenza vaccination program, even in seasons with limited vaccine effectiveness. Also, it captured the impact of several outcomes with different levels of severity.

Given the already mentioned, multiple data source ecological nature of the study; further investigations are warranted, with the perspective of evaluating the sensibility of the approach in other seasons, countries and data sources.

The knowledge on health benefits in terms of influenza-related hospitalizations and deaths averted by the vaccination program will allow better understanding the impact of the national vaccination strategies and strengthening public health communication with the general public and policymakers, to support public health plans towards the increase of vaccine coverage in high-risk groups.

## Supplementary information

**Additional file 1.** Deduction of the formulas of number of averted events (NAE) and number needed to vaccinate (NNV).

**Additional file 2.** Construction of empirical distributions for number of influenza-related events, vaccine coverage (VC) and influenza vaccine effectiveness (IVE) and estimation of confidence intervals for number of averted events (NAE), prevented fraction (PF) and number needed to vaccinate (NNV)

## Data Availability

The Hospital Discharge dataset is not publicly available. The Health Systems Central Administration (Administração Central do Sistema de Saúde, ACSS) provides the Hospital Discharge dataset to the National Health Institute in a regular basis. This dataset is anonymized and may be used for epidemiological studies. The aggregated data used in this specific study is available from the corresponding author upon reasonable request.

## Abbreviations

CI: Confidence Interval
EU: European Union
ICD: International Classification of Diseases
IRE: Influenza-Related events
IVE: Influenza Vaccine Effectiveness
NAE: Number of Averted Events
NNV: Number Needed to Vaccinate
PF: Prevented Fraction
SARI: Severe Acute Respiratory Illness
VC: Vaccine Coverage
