# Supplementary material for: Impact of national influenza vaccination strategy in severe influenza outcomes among the high-risk Portuguese population
Source: BMC Public Health. 2019 Dec 16;19:1690. doi: 10.1186/s12889-019-7958-8 (PMC6916191; doi:10.1186/s12889-019-7958-8)
Supplement: Supplementary file 1 — Additional file 1. Deduction of the formulas of number of averted events (NAE) and number needed to vaccinate (NNV). [file 12889_2019_7958_MOESM1_ESM.docx]

Additional file 1. Deduction of the formulas of number of averted events (NAE) and number needed to vaccinate (NNV)

For measuring the impact of influenza vaccine programme we used the definition of vaccine effects suggested by Halloran (2006) and the review of the impact of vaccination programmes by Hanquet et al. (2013), adapted to influenza vaccination.

The impact of influenza vaccination programmes consists in the comparison of the incidence rate in a population with an influenza vaccination programmes (I_IVP_) in place with a hypothetical totally susceptible population that has never been exposed to the intervention (I_NoIVP_).

The prevented fraction in such situation would be (Eq1):

PF= (I_NoIVP-_I_IVP_)/ I_NoIVP_

where

PF is the prevented fraction;

I_NoIVP_- the incidence in a population with no influenza vaccination programme and

I_IVP_- the incidence in population with influenza vaccination programme

The previous formula is equivalent to (Ep2):

PF x I_NoIVP_ = I_NoIVP-_I_IVP_ ⬄ I_IVP_ = I_NoIVP_ (1- PF) ⬄ I_NoIVP_ = I_IVP_ /(1- PF)

Considering that

I_NoIVP_ =N / Pop where N is the number of influenza cases in the population without influenza vaccine programme (Pop) and

I_IVP =_ n/Pop where n is the number of influenza cases in the population with influenza vaccine programme (Pop)

Equation 2 could be written as (Equation 3):

$$\frac{N}{Pop}= \frac{n/Pop}{(1-PF)}$$

Assuming that both population have the same dimension, Equation 3 could be simplified to (Equation 4):

N= $\frac{n}{(1-PF)}$

The number of averted events (NAE) would be the difference of influenza cases in a population without influenza vaccination programme (N) and with an influenza vaccination programme (n) (Eq 5).

NAE= N- n ⬄ NAE = $\frac{n}{(1-PF)}-n$ ⬄ NAE = $\frac{n}{1-\left( VC x VE \right)}-n$ ⬄ NAE = $n x \frac{VC x VE}{1-\left( VC x VE \right)}$
