# Supplementary material for: Impact of national influenza vaccination strategy in severe influenza outcomes among the high-risk Portuguese population
Source: BMC Public Health. 2019 Dec 16;19:1690. doi: 10.1186/s12889-019-7958-8 (PMC6916191; doi:10.1186/s12889-019-7958-8)
Supplement: Supplementary file 2 — Additional file 2. Construction of empirical distributions for number of influenza-related events, vaccine coverage (VC) and influenza vaccine effectiveness (IVE) and estimation of confidence intervals for number of averted events (NAE), prevented fraction (PF) and number needed to vaccinate (NNV) [file 12889_2019_7958_MOESM2_ESM.docx]

Additional file 2. Construction of empirical distributions for number of influenza-related events, vaccine coverage (VC) and influenza vaccine effectiveness (IVE) and estimation of confidence intervals for number of averted events (NAE), prevented fraction (PF) and number needed to vaccinate (NNV)

To estimate 95% confidence intervals for NAE, NNV, and PF we used Monte Carlo simulations. First, we constructed empirical distributions for all input parameters (number of influenza-related events, vaccine coverage (VC) and influenza vaccine effectiveness (IVE)).

For the number of influenza-related events (count data) we assumed a Poisson distribution.

To obtain empirical distribution of IVE we assumed a normal distribution for *log(1-IVE)*. This option was chosen since IVE estimates are obtained as *IVE=1-Odds ratio* (OR), estimated by logistic regression model. *OR* is obtained as *OR=exp(*$\beta$*)*, where $\beta$ represents coefficient from logistic regression model. We transformed IVE into $\hat{\beta_{ve}}=log(1-\hat{IVE)}$ and IVE 95% confidence interval upper $\hat{IVE(u)}$ and lower $\hat{IVE(l)}$ bounds into $\hat{\beta_{ive(l)}}=log(1-\hat{IVE(u))}$ and $\hat{\beta_{ve(u)}}=log(1-\hat{IVE(l))}$, respectively. We computed a standard error ${SE}_{\beta_{ive}}=\frac{\hat{\beta_{ive(u)}}-\hat{\beta_{ive(l)}}}{2*Z_{0.975}}$, where $Z_{0.975}$ represents a quantile of standard Normal distribution, and generated pseudo-random numbers from Normal distribution:

$\hat{\beta_{ive}}\sim Normal\left( \hat{\beta_{ive}} ,{SE}_{\beta_{ive}} \right).$

Simulated values of $\hat{\beta_{ive}}$ were transformed back to original scale using following formula: $IVE=1-exp(\hat{\beta_{ive}}$).

For VC, since originally VC was estimated from a complex survey and the 95% confidence interval was computed using logit transformation (ref), we transformed VC into $\hat{\beta_{vc}}=log\left( \frac{\hat{VC}}{1-\hat{VC}} \right)$. Lower and upper limits of VC confidence interval were transformed as $\hat{\beta_{vcl}}=log\left( \frac{\hat{VC(l)}}{1-\hat{VC (l)}} \right)$ and $\hat{\beta_{vcu}}=log\left( \frac{\hat{VC(u)}}{1-\hat{VC (u)}} \right)$ to estimate${SE}_{\beta_{vc}}=\frac{\hat{\beta_{vcu}}-\hat{{\beta vc}_{l}}}{2*Z_{0.975}}$, where $Z_{0.975}$ represents a quantile of standard Normal distribution.

We generated pseudo-random numbers from Normal distribution $\hat{\beta_{vc}}\sim Normal\left( \hat{\beta_{vc}} ,{SE}_{\beta_{vc}} \right)$ and applied inverse transformation

$$VC=\frac{exp(\hat{\beta_{vc}})}{1+exp(\hat{\beta_{vc})}}$$

 to return to original scale.

We draw 10 000 samples of number of influenza-related events, IVE and VC and used them to construct empirical distributions of NAE, NNV, and PF. The 2.5 and 97.5 percentiles of these empirical distributions were used as lower and upper limits of the 95% confidence intervals for NAE, NNV, and PF.
